# Systematic approach identifies RHOA as a potential biomarker therapeutic target for Asian gastric cancer

**DOI:** 10.18632/oncotarget.12963

**Published:** 2016-10-28

**Authors:** Hae Ryung Chang, Seungyoon Nam, Jinhyuk Lee, Jin-Hee Kim, Hae Rim Jung, Hee Seo Park, Sungjin Park, Young Zoo Ahn, Iksoo Huh, Curt Balch, Ja-Lok Ku, Garth Powis, Taesung Park, Jin-Hyun Jeong, Yon Hui Kim

**Affiliations:** ^1^ New Experimental Therapeutics Branch, National Cancer Center of Korea, Goyang-si, Republic of Korea; ^2^ Research Institute of Women's Health, Sookmyung Women's University, Seoul, Republic of Korea; ^3^ College of Medicine, Gachon University, Incheon, Republic of Korea; ^4^ Korean Bioinformation Center, Korea Research Institute of Bioscience and Biotechnology, Daejeon, Republic of Korea; ^5^ Department of Nanobiotechnology and Bioinformatics, Korea University of Science and Technology, Daejeon, Republic of Korea; ^6^ College of Pharmacy, Yonsei Institute of Pharmaceutical Sciences, Yonsei University, Incheon, Republic of Korea; ^7^ Animal Sciences Branch, National Cancer Center of Korea, Goyang-si, Republic of Korea; ^8^ Department of Statistics, Seoul National University, Seoul, Republic of Korea; ^9^ Bioscience Advising, Ypsilanti, MI, USA; ^10^ Korean Cell Line Bank, Seoul National University, Seoul, Republic of Korea; ^11^ Cancer Center, Sanford-Burnham Prebys Medical Discovery Institute, La Jolla, CA, USA; ^12^ Discovery Biology, CrystalGenomics Inc., Seongnam-si, Republic of Korea

**Keywords:** gastric cancer, RHOA, G-protein, biomarker, therapeutic target

## Abstract

Gastric cancer (GC) is a highly heterogeneous disease, in dire need of specific, biomarker-driven cancer therapies. While the accumulation of cancer “Big Data” has propelled the search for novel molecular targets for GC, its specific subpathway and cellular functions vary from patient to patient. In particular, mutations in the small GTPase gene *RHOA* have been identified in recent genome-wide sequencing of GC tumors. Moreover, protein overexpression of RHOA was reported in Chinese populations, while *RHOA* mutations were found in Caucasian GC tumors. To develop evidence-based precision medicine for heterogeneous cancers, we established a systematic approach to integrate transcriptomic and genomic data. Predicted signaling subpathways were then laboratory-validated both *in vitro* and *in vivo*, resulting in the identification of new candidate therapeutic targets. Here, we show: i) differences in *RHOA* expression patterns, and its pathway activity, between Asian and Caucasian GC tumors; ii) *in vitro* and *in vivo* perturbed *RHOA* expression inhibits GC cell growth in high RHOA-expressing cell lines; iii) inverse correlation between RHOA and RHOB expression; and iv) an innovative small molecule design strategy for RHOA inhibitors. In summary, RHOA, and its oncogenic signaling pathway, represent a strong biomarker-driven therapeutic target for Asian GC. This comprehensive strategy represents a promising approach for the development of “hit” compounds.

## INTRODUCTION

Gastric cancer (GC) is the fourth-most common cancer in the world, with an estimated 1.034 million new cases in 2015, and the third-highest cause of cancer deaths, estimated at 785,558 in 2014 [1]. GC mortality is highest in East Asia, with over half the world's total deaths, followed by Central and Eastern Europe, and Central and South America [1]. These statistics show clear disease differences based on geographic region, race, and ethnicity [1].

Surgery is the 1^st^ line treatment for GC, producing an overall survival rate of 60 - 70% for early stage disease (www.cancer.gov) [2]. High GC-incidence nations such as Japan and Korea now implement routine screening for early detection, when the disease is highly curable [3, 4]. In many less developed countries, however, GC is mostly detected only in its advanced stages, precluding curative surgical resection and necessitating systematic treatment. The low efficacy of current therapies results in advanced or metastatic GC having a low survival rate of 5–20%, and a particularly poor prognosis for peritoneal GC recurrence [5, 6]. Together, these facts thus reinforce the urgent need for improved biomarker-driven, “targeted” therapeutic strategies.

A disease phenotype is the culmination of complex network interactions between multiple biological processes/pathways [7]. Consequently, “network medicine” can enhance the understanding of the detailed mechanisms and cellular progression of heterogeneous (e.g., gastric, pancreatic, etc.) cancers, revealing better biomarker-driven targets for drug development [7]. This approach, however, requires detailed analyses of multiple signaling transduction pathways, especially in specific tumor subpopulations. Using our previously developed approach, PATHOME [8], an in-depth, computational network systematic analysis, we successfully identified biomarkers for gastric and breast cancer tumor progression [8, 9]. We also predicted GC progression to involve focal adhesion subpathways in, which rely on RHOA for cytoskeletal [8, 10]. RHOA is frequently overexpressed in Japanese and Chinese GC patient tumors [11, 12], while GC datasets from The Cancer Genome Atlas (TCGA) depository [13] showed RHOA mutations, not mere overexpression, in diffuse type GC tumors [14, 15]. Based on RHOA's involvement in other cancers, in this study, we herein investigated whether RHOA inhibitors could successfully be identified as an evidence-based, biomarker-driven therapeutic option for GC patients. In particular, we observed that *RHOA* upregulation, concomitant with reduced *RHOB* downregulation, was a common occurrence in Asian GC tumors. Moreover, RHOA perturbation resulted in strong inhibition of GC cell proliferation and tumor growth. Lastly, we developed an evidence- and hypothesis-driven, cheminformatics approach to successfully identify five candidate RHOA inhibitors. The latter represents a straightforward and innovative method for the development of promising, enzyme-binding small molecules for suppressing oncogenic signaling pathways

## RESULTS

### Identification of *RHOA* upregulation in Asian gastric cancer

**Figure 1:**
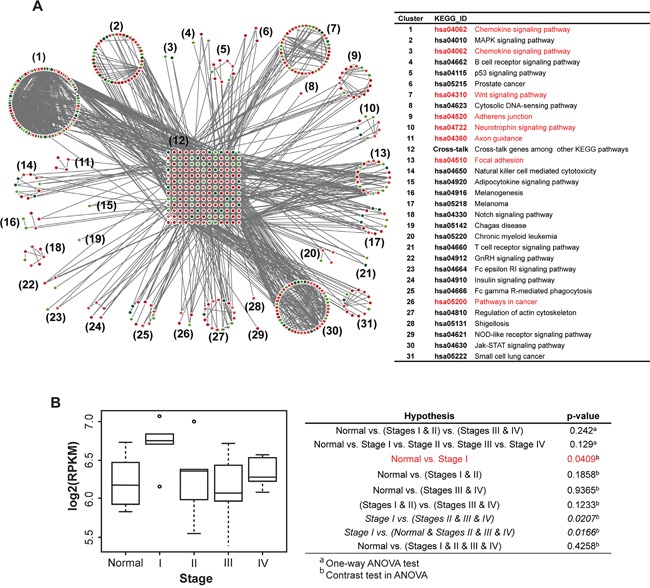
Network analysis in a Korean GC RNA-Seq dataset shows an underlying GC tumor oncogenetic network, under various signaling contexts **A.** PATHOME analysis of Korean GC dataset GSE36968 resulted in 31 functional clusters consisting of significant KEGG subpathways. The clusters were assigned to their corresponding KEGG pathway titles. The network diagram showed upregulated genes in red and downregulated genes in green (left panel), and the designated KEGG pathway titles noted in the right table. The network contained RHOA as a “cross-junction” involved in several pathways (see details in the main text). Pathways related to RHOA are marked red. **B.** From previous Asian GC samples (deposited in GEO; GSE36968), RHOA expression was inspected throughout GC tumor stages. The x-axis represents stage, and the y-axis log_2_-scaled RPKM. Stage I patients showed higher *RHOA* gene expression compared to other stage patients, including normal controls.

In our previously study, we identified focal adhesion pathways as significant to GC by transcriptomic analysis using PATHOME [8]. Use of an independent Asian RNA-seq dataset [GEO accession: GSE36968 (24 GC, 6 normal samples) [16] validated our previous finding by showing RHOA association with actin cytoskeleton signaling, one of the top 31 pathway clusters (Figure 1A). In particular, we show here that chemokine signaling, focal adhesion, and other cancer-related (Cluster 6, 17, 20, 26 and 31) pathways (Figure 1A, right panel), all involve RHOA. Using the same dataset, we showed *RHOA* expression levels by tumor stage (Figure 1B; see sample information in Supplementary Table S1), revealing statistically significant (p-value 0.0409 by contrast in one-way ANOVA) association with Stage I tumors (see Supplementary Table S1), as compared to normal stomach (Figure 1B).

**Figure 2:**
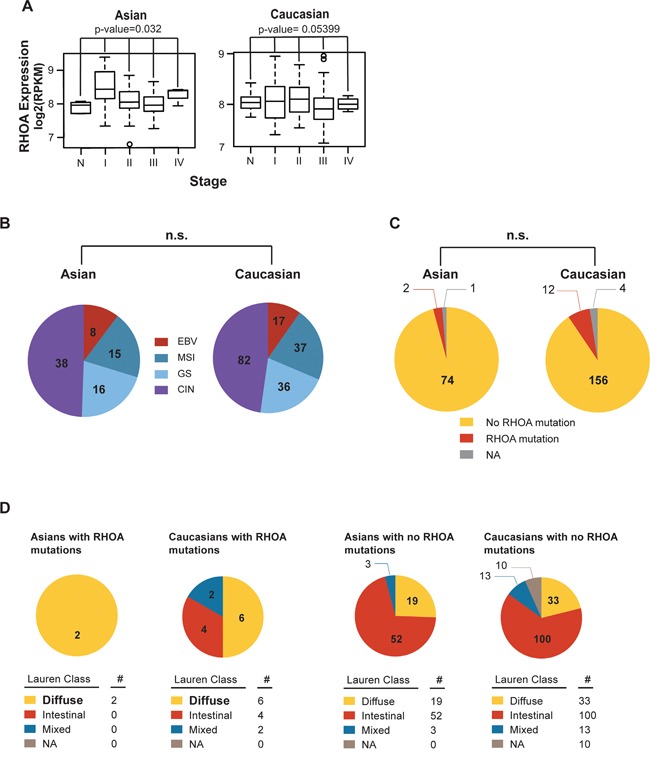
*RHOA* expression analysis shows difference in Asian vs. Caucasian **A.** *RHOA* mRNA expression levels, by cancer stage in both Asian and Caucasian races, showed *RHOA* expression to significantly associate (p-value 0.032 by one-way ANOVA) with Asian GC disease stages, but not in Caucasians. In particular, *RHOA* up-regulation in Stage I, compared to normal, is shown. **B.** molecular subtypes between TCGA Asian and Caucasian GC patients. By using cBioPortal (data version: Stomach Adenocarcinoma (TCGA, Nature 2014)), the proportions between the two races, in terms of molecular subtypes, were not statistically different. **C.** *RHOA* mutation between TCGA Asian and Caucasian datasets. By using cBioPortal (as above), the proportions between the two racial groups, in terms of *RHOA* mutations, were not statistically different. **D.** *RHOA* mutations, as compared between TCGA Asian and Caucasian data, according to Lauren class. Diffuse type was bolded to show enrichment of mutation compared to intestinal and mixed. As shown, the proportions between the two ethnicities in terms of *RHOA* mutations and Lauren class were not statistically different.

Using the TCGA GC dataset [13], we next compared *RHOA*-related gene expression patterns between 77 Asian and 172 Caucasian cases (Figure 2). In Asian GC, *RHOA* expression showed significant differences between disease stages (p-value 0.032 by ANOVA test) (Figure 2A; see sample information in Supplementary Table S1). Also, for Figure 2A, we performed another statistical test, 1,000 random samplings without replacement. In each random sampling, we permuted stage labels against the original data, subsequently calculating F-statistic. After 1,000 random samplings, we obtained the distribution of F-statistic. For example, if the observation of F-statistic for the original data as *f_obs_*., the empirical p-value was obtained by Pr(F > *f_obs_*). As a result, the empirical p-values for Asian and Caucasian were 0.030 and 0.054, respectively.

No significant differences were seen between the two groups with regard to the molecular subtypes characterized by TCGA (*e.g.*, Epstein-Barr virus, microsatellite instability, genome stability, and chromosome instability) (Figure 2B). 2 of the 77 Asian GC tumors (2.5%) showed *RHOA* mutations, as did 12 of the 172 Caucasian tumors (7.0%) (Figure 2C, 2D). Due to the limited number of *RHOA* mutations, the lack of significance should be carefully interpreted. Thus, from our comparison of the Asian vs. Caucasian datasets, we observed significant Asian GC *RHOA* upregulation, allowing us to proceed further to identify key genes in the *RHOA*-associated actin cytoskeleton signaling pathway.

### GC cell lines shows RHOA expression level-dependent growth inhibition upon RNAi gene knockdown

**Figure 3:**
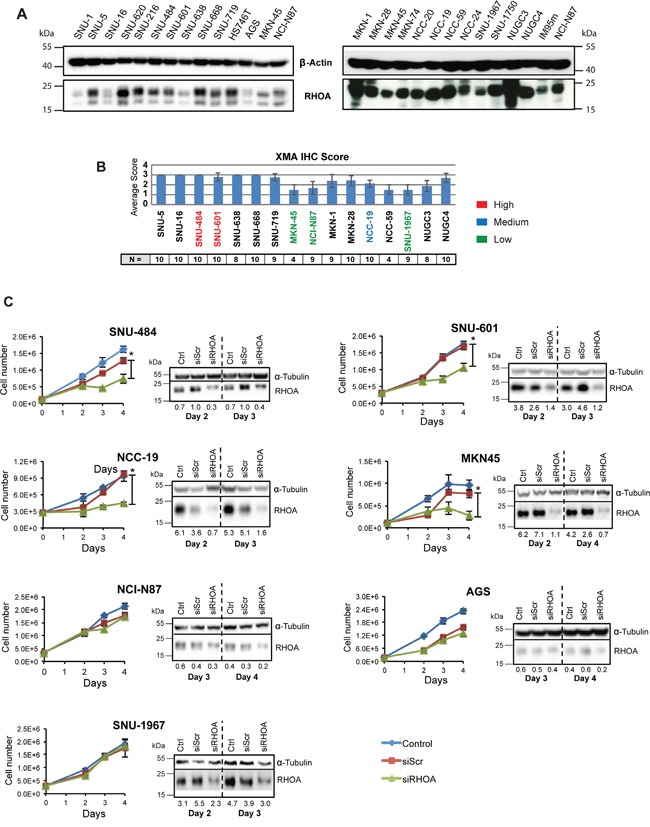
Gene knockdown of *RHOA* in GC cell lines show differences in cell proliferation RHOA protein expression evaluation on GC cell line panel shows various levels of protein expression. **A.** Western blot analysis of RHOA expression on a 26-GC cell line panel. **B.** Immunohistochemistry against RHOA of tissue microarray of cell lines that were successfully established into xenograft models graded from 0 – 3. (2.5~3: high RHOA-expressing cells-red; 2~2.5: medium RHOA-expressing cells-blue; <2: low RHOA expressing cells-green). N is the number of repeats per cell line. **C.** cell proliferation results after siRHOA gene knockdown. Harvested cells were mixed with Trypan blue (1:1) and live cells were counted. High *RHOA-*expressing GC cell lines, such as SNU-484 and SNU-601, showed decreased cell proliferation, whereas the *RHOA*-low expressing cell line SNU-1967 showed little effect (asterisks (*) indicate p-values < 0.05). Western blot analysis showed a decrease in RHOA protein after siRHOA infection of numerous GC cell lines.

In addition to our tumor studies described above, we examined RHOA signaling, as a potential therapeutic target, in living cells. For this purpose, RHOA protein expression was assessed in 25 GC cell lines. As shown in Figure 3A and 3B, high-to-medium RHOA expression levels were observed in a majority of GC cells examined. Those results are consistent with our immunohistochemistry (IHC) studies (Figure 3B). IHC RHOA expression levels were ranked as follows: 2.5–3.0 as high-, 2.0–2.5 as medium-, and grades ≤2.0 as low-expression. Thus, SNU-484 and SNU-601 GC cells were classified as high-expressing, NCC-19 as medium-expressing, and AGS, NCI-N87, MKN45, and SNU-1967 as low-expressing cell lines (Figure 3A and 3B).

To further study RHOA function in GC cells, we performed siRNA knockdown and observed cell viability in the 7 cell lines mentioned above. Growth inhibition directly correlated with the level of *RHOA* knockdown. For example, poorly knocked down GC cell lines (e.g., AGS, NCI-N87 and SNU-1967), as confirmed by Western blot analysis, showed less growth inhibition than those with *strong RHOA* knocked down (e.g., SNU-484, SNU-601, MKN45 and NCC-19 cells) (Figure 3C). Except for MKN45 cells, the high RHOA-expressing cell lines showed greater si*RHOA* knockdown, and were not viable, while the low-expressing cell lines were less sensitive to si*RHOA* knockdown. AGS cells however, were sensitive to the transfection reagent (data not shown), thus precluding their further analysis.

**Figure 4:**
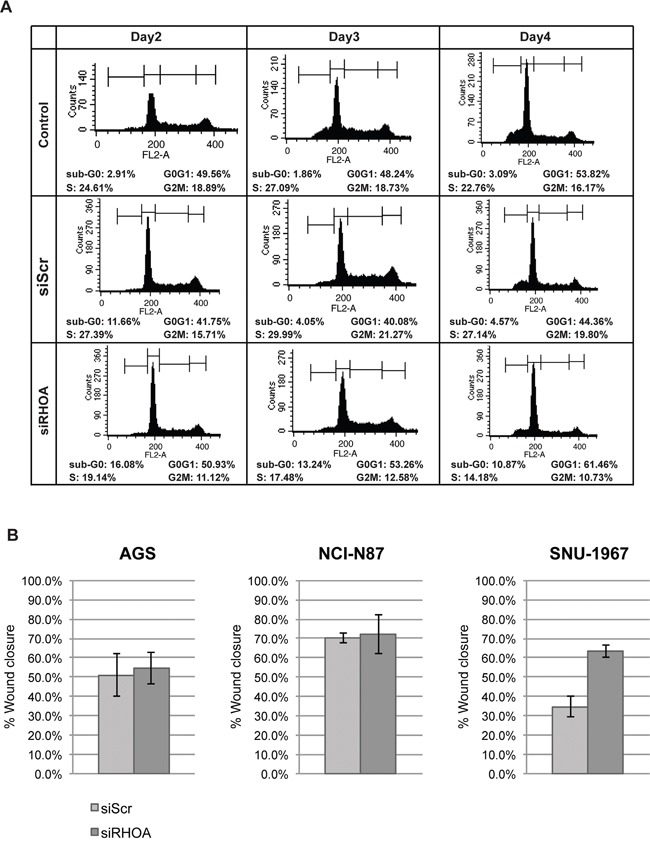
Gene knockdown of *RHOA* in GC cell lines show differences in cell function depending on RHOA protein expression **A.** cell flow cytometry results showed that sub-G0 population (apoptotic) increased as *RHOA* was knocked down upon *RHOA* knockdown in SNU-601 cell line. **B.** Migration assay was performed on three low RHOA-expressing GC cell lines (AGS, NCI-N87 and SNU-1967) after RHOA knockdown by siRNA. AGS and NCI-N87 show little difference in migration between siScr and siRHOA, and SNU-1967 show increased migration upon RHOA knockdown.

We also examined cell cycle distribution, showing that most si*RHOA* treated cells exhibited increased apoptosis (i.e., sub-G_0_ cellular debris), in positive correlation with *RHOA* knockdown (Figure 4A and Supplementary Figure S1). Although si*RHOA* knockdown minimally inhibited growth of some RHOA low-expressing cells, it might possibly hinder actin-related cell functions such as migration. To assess the effect of *RHOA* knockdown on the migration phenotype, we performed “wound healing” assays on the three (AGS, NCI-87 and SNU-1967) low RHOA-expressing cell lines that had reduced proliferation upon further *RHOA* knockdown. Loss of migration was not observed in any of the three cell lines when comparing si*Scr* vs. si*RHOA* treatment (Figure 4B). Interestingly, *RHOA* knockdown increased migration in SNU-1967 cells to 63.3% wound closure, compared to 34.7% closure for si*Scr*-transfected cells. These results show that cell lines with differential endogenous RHOA expression differ in their responses to *RHOA* knockdown. Namely, cell growth was more inhibited in cell lines with higher RHOA expression, than in cell lines with low expression, with the latter also demonstrating impaired migration, upon *RHOA* knockdown.

### *shRNA* knockdown of *RHOA* inhibits tumor growth in GC xenograft models

**Figure 5:**
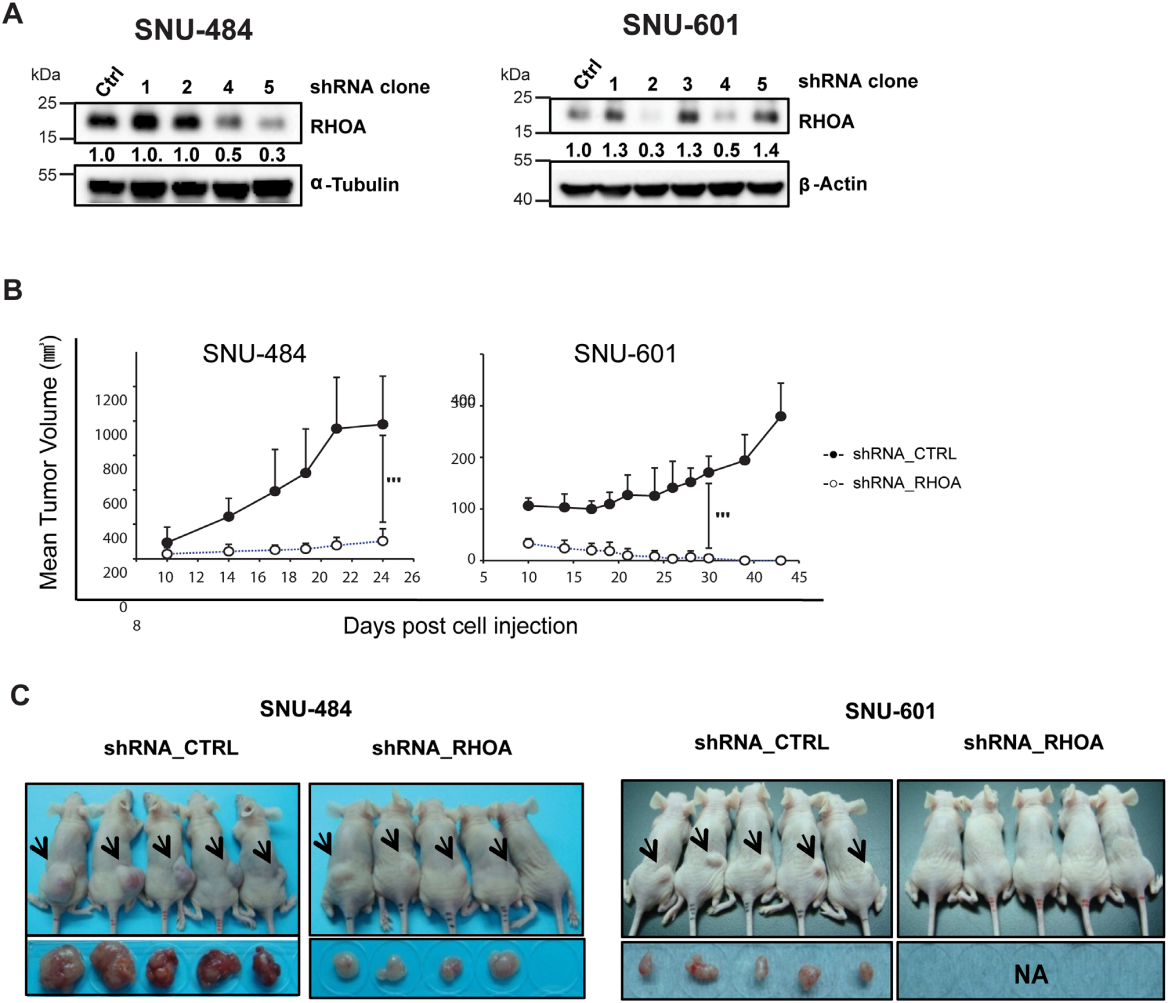
In vivo xenograft model of shRHOA shows decreased tumor size **A.** Western blot analysis shows different knockdown efficiencies of each clone after lentiviral infection of *RHOA* shRNA. **B.** tumor growth was measured from day 10 of inoculation and was monitored until the animals were sacrificed. Tumor growth suppression was observed in *RHOA* shRNA knockdown tumors (SNU-484 and SNU-601), as compared to the various control groups. *** p-value: SNU-484p-value = 0.0061, SNU-601 p-value = 0.0004) **C.** photograph of mice bearing xenograft tumors. Arrows indicate the location of the tumors. Tumor sizes from the *RHOA* knockdown cells were smaller in SNU-484 GC cells, and failed to grow in SNU-601 cells.

To observe *RHOA* knockdown effect *in vivo*, SNU-484 and SNU-601 cells were selected to generate stable cell lines having shRNA-knocked down *RHOA*, for subsequent mouse xenograft studies. Figure 5A shows, by Western blot, different knockdown levels of distinct clones shown by Western blot. A mixture of the greatest RHOA-knock-down (lowest RHOA expressing) cells (clones #4 and #5 for SNU-484 and #2 and #4 for SNU-601) was engrafted to two groups of nude mice with control- or *RHOA* shRNA-transfected SNU-484 and SNU-601 cells. Tumors from *RHOA* knockdown cells grew just above the baseline, showing significant differences in size, as compared to the control tumors (p-value < 0.05; Figures 5B and 5C). In SNU-601 xenografts, shRNA-RHOA tumor growth was completely inhibited. Overall, of the two GC xenograft models, *RHOA* knockdown suppressed tumor growth in both, reaffirming its role in GC oncogenesis.

### RHOA RNAi knockdown shows RHOA-RHOB potential crosstalk

**Figure 6:**
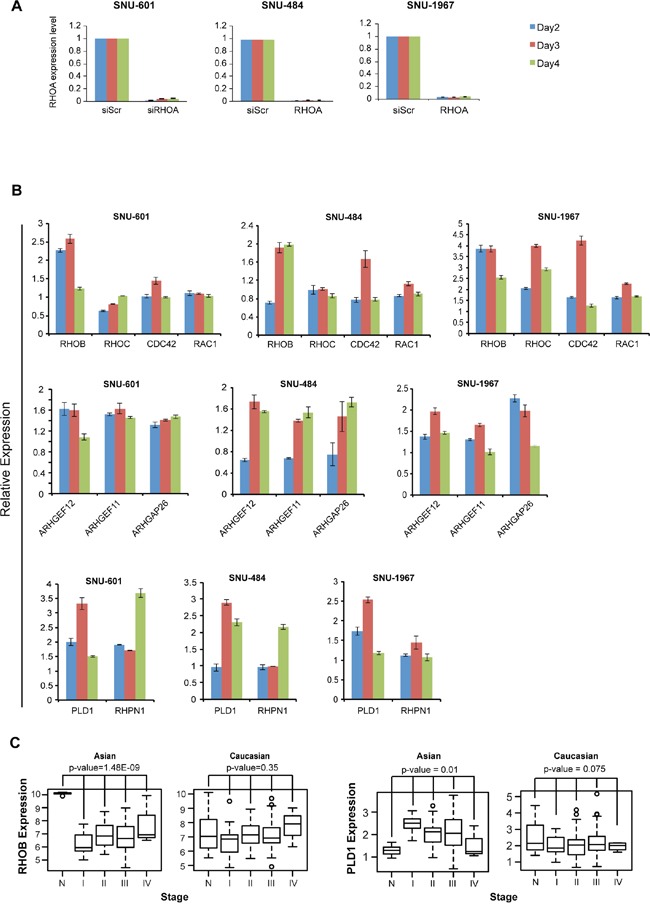
*RHOA* knockdown results show unique patterns of downstream genes and induction of cell death **A.** quantitative RT-PCR results of SNU-601 cells after *RHOA* siRNA knockdown. *RHOA* was successfully knocked down in RHOA high-expressing (SNU-484 and SNU-601) as well as low-expressing (SNU-1967) cell lines. **B.** mRNA expression level of various RHOA network genes were evaluated by RT-PCR after siRNA knockdown of RHOA. RHOB was consistently upregulated in 3 cell lines (SNU-601, SNU-484 and SNU-1967), but not other Rho family genes *RHOC, CDC42* and *RAC1*. Upstream genes *ARHGAP26, ARHGEF11* and *ARHGEF12* were found upregulated in all three cell lines, as well as *PLD1*, and to a lesser degree, RHPN1. **C.** *RHOB* is significantly downregulated in Asian, but not in Caucasian, GC (p-values by one-way ANOVA), and *PLD1* shows upregulation in Asian GC. Gene expression was inspected with reference to normal samples and tumor stages.

We next explored the subpathway(s) involved in GC cell growth inhibition upon si*RHOA* transfection, by assessing gene expression levels were evaluated by RT-PCR. Two (SNU-484 and SNU-601) RHOA high-expressing cell lines and one (SNU-1967) low-expressing cell line (Figure 6A) we used. In all three cell lines, the *RHOA* upstream genes, *ARHGEF11*, *ARHGEF12* and *ARHGAP26* were upregulated (Figure 6B), as was a downstream gene, *PLD1*.

Interestingly, *RHOB*, a Rho family gene and homolog of RHOA, was consistently upregulated in all three cell lines (Figure 6B). Overall, the expression levels of RHOA pathway genes were upregulated in SNU-1967 (≥2-fold), compared to SNU-484 and SNU-601 cells (Figure 6B). In the GC dataset as mentioned above, *RHOB* was significantly downregulated in tumor samples compared to normal tissues in Asian GC, but not in Caucasian GC tumors (Figure 6C). Conversely, the phospholipases gene D1 (*PLD1*) was upregulated in Asian, but not in Caucasian GC samples. These findings also showed that the expression levels of several *RHOA*-related genes were altered upon mRNA perturbation.

We also performed another statistical measurement (for Figure 6C) by using 1,000 random samplings without replacement, for *RHOB* and *PLD1*, as described above. For *RHOB*, the empirical p-values for Asian and Caucasian were 0.000 and 0.361, respectively. And, for *PLD1*, the empirical p-values for Asian and Caucasian were 0.009 and 0.077, respectively. These empirical p-values of *PLD1* and *RHOB* agreed with the p-values results of ANOVA tests.

### *In Silico* screening of small molecules specifically binding to RHOA

**Figure 7:**
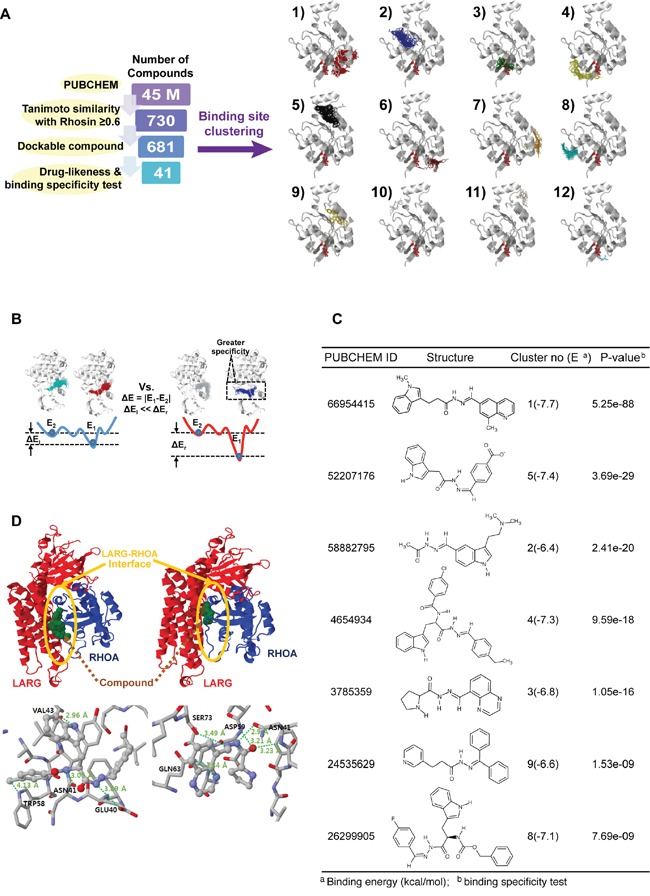
A systematic search of RHOA inhibitor small molecules suggests several candidates **A.** number of compounds remaining after each round of filtering (left panel). The selected compounds were docked in different clusters of novel RHOA binding pockets (right panel). **B.** schematic binding-specific test figure. Smaller (left), and larger specific situations (right). E_1_ and E_2_ are the first and the second representative energies in binding clusters. Larger energy differences (ΔE_r_) represent more specific compounds. **C.** chosen RHOA candidate drug list tabulated by PUBCHEM ID, structural figure, cluster number and binding energy in parenthesis, and P-value calculated by binding-specific test. **D.** structure figures in cluster no. 4 (left) and no. 3 (right). (Upper panel) ARHGEF12 (LARG) and RHOA are drawn by red and blue cartoon images, respectively. The LARG-RHOA interfaces are denoted by the yellow circles. The bound compounds of clusters no. 4 and no. 3 are drawn by green spheres (lower panel). Hydrogen bonds, depicted by thick dotted lines with the distance between donor and acceptor atoms. Interacting residues with the compound are expressed by residue names and respective numbers.

Our overall procedure for our virtual screening of target RHOA inhibitors is shown in Figure 7A. First, we searched PUBCHEM (pubchem.ncbi.nlm.nih.gov) [17] for compounds structurally similar to the known RHOA inhibitor, Rhosin [18], to measurement similarity (Tanimoto score). We further selected all compounds dockable to the mapped binding regions in the RHOA crystal structure. The potential binding regions defined as “clusters,” for the dockable compounds, are shown in the right panel in Figure 7A. Finally, we applied binding specificity as well as drug-like physicochemical properties (Lipinski's rule of five) [19] for further filtering (see Materials and Methods).

Of the 45 million compounds in PUBCHEM, we identified 41 with high *RHOA-*binding specificity (Figure 7A) based on the energy differences between the first and second binding energies of the compound (Figure 7B; details in Supplementary Methods). Due to the small volume of some of the compounds compared to the potential binding pocket volume, non-specific binding to multiple regions may occur even within the pocket. Consequently, the binding energy differences between these regions within the pocket were calculated to identify compounds with the greatest energy differences. These were then selected as specifically binding to a certain region, corresponding to the first and the second lowest-binding energies, referring to the effective binding specificity to the top region within any specific cluster.

From the 41 compounds, 7 representatives were chosen and tabulated (Figure 7C). Since the protein ARHGEF12 (LARG) physically binds to RHOA at the protein-protein interaction interface [20], we focused on this region to identify two compounds (PUBCHEM IDs 4654934 and 3785359) that bound to clusters 4 and 3, respectively (Figure 7D). These two compounds could, computationally, directly bind to the protein-protein interaction interface of the LARG-RHOA complex. Another five compounds (PUBCHEM IDs 66954415, 58882795, 52207176, 26299905 and 24535629, binding to cluster numbers 1, 2, 5, 8 and 9, respectively) were found on other surfaces (i.e. not in the LARG-RHOA interface). Thus, these two inhibitors identified above may have an allosteric effect, indirectly preventing LARG-RHOA interaction by altering the RHOA conformation.

### Synthesis and biological evaluation of potential inhibitors targeting RHOA

**Figure 8:**
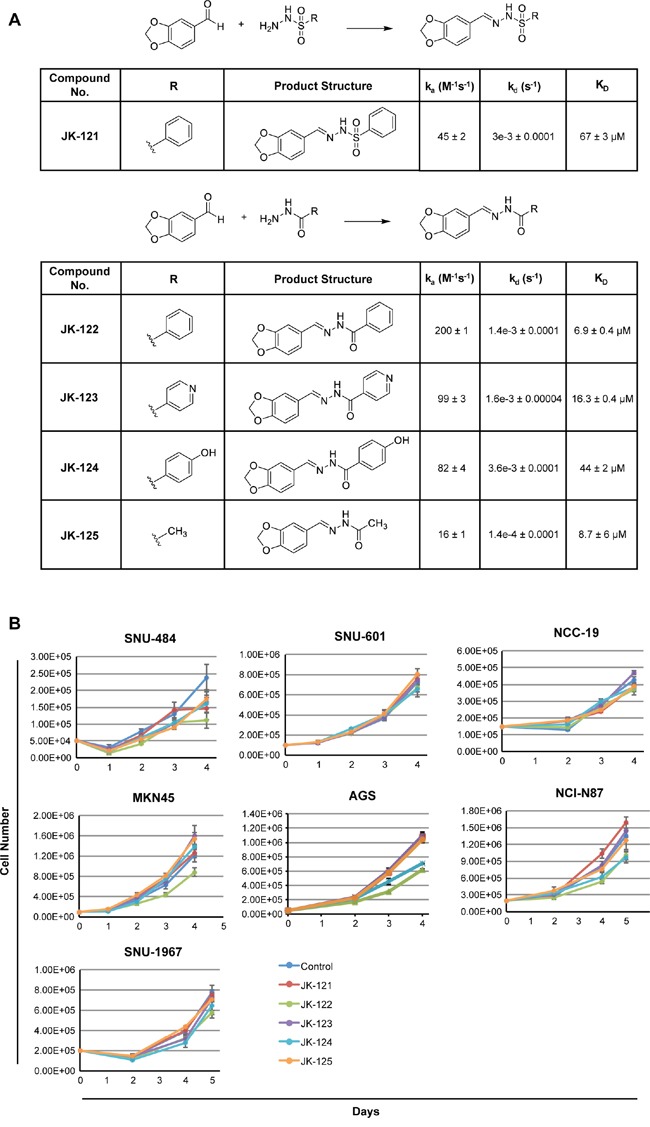
Small molecule inhibitor RHOA candidates inhibit GC cell growth **A.** scheme and structure of the five candidates. Reagents and conditions: MeOH or EtOH, RT or reflux, 2-40 h. On and off rates as well as binding constant (K_D_ was determined by surface plasmon resonance. **B.** 7 GC cell lines, SNU-484, SNU-601, NCC-19, MKN-45, AGS, NCI-N87 and SNU-1967 were treated with the five small molecular candidates. GC cell lines showed growth inhibition when treated with 20 μM of JK-122.

Considering our computational and laboratory results, we focused on rigorous design of hydrazide homologues, a functional group found in several of the successfully identified compounds. Based on the results from our *in silico* screening, we designed a hydrazide functional group serving as a spacer skeleton, with the structural variation of the R moiety shown in Figure 8A. For greater diversity, we rationalized that structurally similar compounds would likely exhibit similar biological activity, and we thus fixed the piperonyl group and hydrazide spacer moiety in the compound structure. By varying the R moiety for structural modification, we expected different biological activities, depending on the moiety's coverage. For the purpose of achieving wide diversity by navigating through the relevant chemical space, five distinct compounds, representing different chemical properties, were designed and synthesized. Of these, the compound JK-122 was synthesized to evaluate the activity of a non-polar phenyl group series as an R moiety. Compared to JK-122, JK-121 was synthesized to evaluate the effect of a sulfonyl functional group series in the hydrazide spacer. JK-123 and JK-124 were synthesized to assess the necessity and the activity of a hydrophilic functional group series, such as the nitrogen or the hydroxyl group, to participate in hydrogen bonding. JK-125, by contrast, was synthesized for assessing the effect of replacing an aromatic group with an aliphatic group. The synthetic strategy by piperonal treatment of selected hydrazides for the preparation of compounds JK-121~125, is depicted in Figure 8A.

Protein-small molecule interactions were further analyzed by surface plasmon resonance (SPR) to determine the binding affinity as well as the on/off-rate of each compound to RHOA protein. Of the five compounds tested, JK-122 showed the lowest dissociation constant, K_D_ at 6.9±0.4 μM (Figure 8A) and k_d_ (dissociation rate or ‘off rate’) of 1.4e-3±0.0001 s^-1^, both acceptable values for protein binding of small molecule. Thus, of the five compounds we tested, the kinetics data showed JK-122 to be the best for further investigation as a RHOA inhibitor.

For *in vitro* compound assessments, 7 GC cell lines (selected based on high, mid or low-RHOA expression levels) were treated with 20 μM of JK-121~ 125. JK-122 showed up to 50% growth inhibition at 20 μM, while the others had little to no effect (Figure 8B). A previous Rhosin identification study reported that an aromatic ring on both ends of the molecule may be necessary, and our results confirm this [18]. Thus, our synthetic strategy and these “lead” compounds may provide valuable information for further optimization of novel RHOA inhibitors.

## DISCUSSION

In this study, we systematically assessed the role of *RHOA* pathway dysregulation in GC, and the feasibility of clinically targeting RHOA for GC therapy. Our results suggest *RHOA* as a genomic (mutation, amplification, etc.) and transcriptomic (overexpression) biomarker and therapeutic target in Asian GC patients. By showing *RHOA* upregulation as predominant in Korean GC tumors, and mutation more prevalent in Caucasian GCs, we demonstrate GC to have racial-specific etiologies [21]. Specifically, differences in the expression of *RHOA* and its homolog, *RHOB*, implicate Rho GTPases in differential GC (Figure 6). Such findings will ultimately be important for precision medicine, as biomarker-driven cancer therapies inevitably rely on pathway differences for patient stratification. In the short term, however, such knowledge could be valuable for diagnostic purposes.

The finding of variable *RHOA* expression in diverse GC cell lines, is not surprising, considering the high degree of heterogeneity of this specific tumor type [21]. Proper cell line selection is critical for drug target preclinical studies, as drug efficacy may differ between cell lines (and tumor subpopulations), preventing missed “druggable” targets. For example, for preclinical evaluation of trastuzumab, NCI-N87 and OE19 were the only two HER2-overexpressing GC cell lines reported by Wainberg *et al.* [22]. Assessment of diverse cell lines could be highly valuable in clinical settings, such as patient stratification based on oncogenic signaling [23]. Here, we evaluated the downstream effects of *RHOA* knockdown in two *RHOA* high-expressing GC cell lines, SNU-484 and SNU-601, showing different expression patterns, compared to SNU-1967, a *RHOA* low-expressing cell line (Figure 6). This result implies that the RHOA signaling pathway may yield distinct phenotypes, depending on pathway gene expression levels or connection to other pathway networks (i.e. “crosstalk”) [24]. Consequently, the detailed mechanisms of RHOA pathway activators and inhibitors need further investigation, with proper cell line selection for distinct druggable targets, relying upon diverse, physiologically relevant disease models for accurate results.

Upon *RHOA* knockdown, the apoptotic gene *RHOB* [24, 25], was consistently upregulated. It is possible that such anti-correlation occurs downstream of RHOA-RHOB, or depends on an independent subpathway. Thus, it is unclear which downstream genes are up-/down-regulated by *RHOA* knockdown, and further investigation of network genes is necessary to validate those subpathways. While crosstalk between these two genes remains unclear, decreased cell growth in knocked-down *RHOA* high-expressing cells was accompanied by a compensatory increase of *RHOB*, inducing apoptosis, as previously shown [24, 25]. However, while *RHOB* was downregulated in Asian GC tumors, it remained unchanged in Caucasians (Figure 6C; see sample information in Supplementary Table S1). Moreover, the si*RHOA*-knocked down RHOA low-expressing cell line, SNU-1967, showed minimal growth inhibition, even while *RHOB* was upregulated (Figure 6B). Consequently, more study is needed to understand the reciprocal relationship between RHOA and RHOB expression.

RHOA is also a known mediator of the epithelial-to-mesenchymal transition (EMT) [26, 27]. This process is necessary not only for metastasis, but also for single tumor cell peritoneal dissemination that often occurs in metastatic gastric and ovarian cancers [28, 29]. Functionally, RHOA is a small GTPase, which assumes an active form when bound to GTP, and an inactive form when GTP is hydrolyzed to GDP by GTPase-activating proteins [30]. The active form is then restored by transfer of a phosphate group from guanine nucleotide exchange factors (GEFs). The active GTP-bound RHOA triggers activity of its downstream effectors, including mediators of EMT, upon activation of GEFs by numerous metastasis-associated cytokines such as TGFβ, epidermal growth factor, pro-inflammatory factors, and integrins [27, 31–33]. RHOA has also facilitates microtubule remodeling [34], and taken together, these results strongly support the possible efficacy of its therapeutically targeting of the RHOA oncoprotein. To that end, various small molecule RHOA inhibitors are now being examined, including Rhosin and CCG-1423, an inhibitor of RHOA downstream transcription [18]. Here, we also report an innovative strategy for rationally designing RHOA inhibitors.

In summary, we demonstrate involvement of the oncogenic signal mediator RHOA, in gastric cancer, likely via EMT-related cytoskeletal remodeling necessary for cell motility and changes in morphology [31]. This study also supports the importance of pathway construction for developing biomarker-driven cancer therapies. Our analysis clearly shows that RHOA's involvement in GC etiology differs between racial groups, and possibly even between patients. We believe our systematic approach, using distinct cell lines, will greatly contribute to patient stratification, based on RHOA and other pathway genes’ expression. In conclusion, our study establishes RHOA inhibition as a potential treatment for Asian GC patients, warranting further investigation with higher statistical power, and assessment of various small molecule RHOA inhibitors, for the therapy of GC.

## MATERIALS AND METHODS

### Systems biology analysis

PATHOME [8] was used to identify statistically significant subpathways (between GC tissues and normal tissues) (p-value cutoff of 0.05) from pathways of the entire KEGG [35] signaling network database based on our previously studied Korean GC RNA-Seq dataset [GEO accession: GSE36968] [16]. This dataset consisted of 6 normal gastric tissues and 24 GC tumors, and the network consisted of 559 nodes and 2,031 edges. From that specific network, we inspected all known RHOA-downstream genes, finding 63 that were consistently upregulated in two independent GC patient datasets [GEO accessions: GSE13861, GSE27342] [36, 37].

### TCGA GC dataset analysis for Asians and Caucasians

CBioPortal [38] and the UCSC Cancer Genomics Browser (CGB) [39] were used for analysis. GC and normal sample identifiers for both Asians and Caucasians were obtained from the “TCGA, Nature 2014” data version in CBioPortal [38]. The “TCGA_STAD_exp_HiSeq-2015-01-28” data version in the UCSC CGB was used to extract gene expression data for both GC and normal samples for each race.

### *In silico* approach for identifying 730 RHOA small molecule compounds

For detailed methods, refer to “Supplementary Methods.” In brief, based on the structure of the known RHOA inhibitor Rhosin [18], we used Tanimoto similarity scores to search the PUBCHEM database [40], using the program Open Babel [41]. From approximately 45 million compounds, we found 730 similar backbone compounds with high Tanimoto scores of 0.6. Docking software, AutoDock Vina [42] was used, and center of mass (COM) was calculated using calculated using CHARMM [43].

### Lipinski's rule application to the 730 compounds

For detailed methods, refer to “Supplementary Methods.” For further details, refer to “Supplementary Methods.”

### Specific binding test

The significance of the energy differences for all the candidate compounds was calculated using the statistical method used in DEGseq [44]. For detailed methods, refer to “Supplementary Methods.”

### Small molecule synthesis

For detailed methods, refer to “Supplementary Methods.”

### Cell culture

The following human GC cell lines were used within 6 months of tissue resuscitation: NCI-N87, AGS (ATCC), MKN45 (RIKEN), SNU-484, SNU-601, and SNU-1967 (KCLB), cultured in RPMI-1640 (HyClone) and 10% fetal calf serum (HyClone) at 37°C under 5% CO_2_. Cell line identities were validated by short tandem repeat profiling (ATCC, RIKEN, and/or KCLB).

### Short hairpin RNA silencing and mouse xenograft *RHOA*-knockdown model

MISSION^®^ short hairpin RNAs (shRNAs) lentiviral particles (Sigma-Aldrich) were used to stably infect SNU-484 and SNU-601 cells (shRNA empty vector or *RHOA* shRNA), followed by cell culture in 12-well plates using RPMI-1640 plus 10% FBS, with selection by 0.3 mg/mL puromycin (Sigma-Aldrich). Western blotting was used to validate decreased *RHOA* expression, and clones with the least *RHOA* expression were harvested for injection intomice. Approximately 5-7 x 10^6^ cells in log-growth phase were suspended in 0.1 ml phosphate buffered saline (PBS), and subcutaneously injected into the flanks of severe combined immunodeficient (*scid*) mice (Orient Bio). Animals were weighed weekly and tumor diameters measured twice weekly at right angles (d_short_ and d_long_) with electronic calipers, with conversion to volume by the formula V = [(d_long_) x (d_short_) x (d_long_)/2]. When the tumors reached volumes reached 150 and 300 mm^3^, the mice were randomly stratified into two groups of 8 animals, with approximately equal mean tumor volumes.

### Small interfering RNA transfection

40 nM of anti-RHOA (siRNA-RHOA) small interfering RNA (siRNA) SMARTpools, and non-targeting, scrambled control sequences (Dharmacon/GE Healthcare), were used to transfect cells, using DharmaFECT1 transfection reagent (Dharmacon/GE Healthcare). Transfection media was replenished with fresh media after 24h, and the cells then cultured in 5% CO_2_ at 37°C, up to 4 days. Gene knockdown was confirmed by RT-PCR.

### Western blot

Cells were washed twice with PBS, lysed in 20mM Tris pH 7.4, 250mM NaCl, 2mM EDTA, and 1% Triton X-100 buffer, and centrifuged. Supernatants were collected, total protein concentrations determined using BCA protein assay (Pierce), and subjected to PAGE and immunoblotting using anti-RHOA (ab54835, Abcam), anti-β-actin (clone 4967, Cell Signaling), and anti-α-tubulin (clone 05-829, Millipore) antibodies, in conjunction with anti-rabbit (#7074S, Cell Signaling) and anti-mouse (#7076S, Cell Signaling) secondary antibodies. Antibody-bound blots were then visualized by enhanced epichemiluminescence, and quantified using Image Lab software (Bio-Rad).

### Cell migration assay

Radius™ 24-Well Cell Migration Assay kits (Cell Biolabs) were used according to the manufacturer's protocol. 0.5 x 10^5^ (AGS) or 2.5 x 10^5^ cells (NCI-N87 and SNU-1967) cells were then plated and incubated for a 24-hr attachment. siScri and si*RHOA* was transfected using DharmaFECTI (Dharmacon/GE Healthcare) and non-treated control. Each condition was done with n=3 - 4. After 24 hr incubation, the media was removed, and 500 μl fresh media then added to each well. Picturess were taken at 0, 5 and 8 hr intervals, using an Olympus IX70 microscope under brightfield. Migration was observed until about 50% wound closure (AGS: 12 hr; NCI-N87: 28 hr and SNU-1967: 30 hr), and at endpoint, pictures were taken after cells were stained using Cell Staining Solution. Migration edges and wound area was analyzed using Image J (NIH) and Cell Profiler (Broad Institute). % wound closure was calculated by measuring the wound area at 0 hr (A_0_), and measuring the wound area at the end-point (A_T_), and calculating (A_0_ – A_T_) x 100%.

### Real time-PCR

Total RNA was isolated from cell lysates using Isol-RNA Lysis Reagent (5Prime). After lysis, 0.2 ml chloroform was added, the samples shaken for 15 seconds, and then centrifuged at 13,000 rpm for 15 min at 4°C. After centrifugation, the upper layers were placed into new tubes, and 0.5 ml isopropanol was added. The samples were mixed gently, incubated at RT for 10 minutes, and re-centrifuged at 13x1000 rpm for 10 minutes at 4°C. Supernatants were removed and 1 ml of 70% EtOH were added to the pellets, followed by centrifugation at 7500 rpm for 5 minutes at 4°C. The RNA pellets were then dried and dissolved in DEPC-treated water. cDNA was synthesized using ReverTra Ace^®^ qPCR RT Master Mix with gDNA Remover kits (Toyobo). RT-PCR was performed using a CFX384 system (Bio-Rad) and iQ™ SYBR® Green Supermix (Bio-Rad) using primers designed by Primer-BLAST (http://www.ncbi.nlm.nih.gov/tools/primer-blast/) or GenScript (www.genscript.com). Relative expression levels were normalized to GAPDH of the siScr sample corresponding to the day of sampling, using the 2(-delta-delta C_T_) method [45]. All measurements were performed in triplicate.

### Cell cycle analysis by flow cytometry

Negative control cells and cells transfected with siRNA-non-targeting sequences or siRNA-*RHOA* were harvested on days 2, 3 and 4. Collected cells were centrifuged at 1200 rpm for 10 minutes, and washed with 10 ml PBS. After centrifuging, PBS was removed and cold 80% EtOH was added drop-wise to the well-suspended cells. After centrifugation and removal of EtOH, the cells were incubated in 1 ml propidium iodide (PI; 50 μg/ml) with RNase A (0.1 mg/ml) for 30 minutes at 37°C, centrifuged to remove the unbound PI, and resuspended in PBS for cell cycle analysis using a FACS Calibur (BD Biosciences) flow cytometer.

### RHOA binding of small molecule candidate inhibitors

Surface Plasmon Resonance (SPR) was used to study the binding of RHOA protein to synthesized small molecules. Reichert SR7500DC system was used, and RHOA (SRP5127, Sigma Aldrich) protein was immobilized on CMDH gold chip (Reichert) at <7 μg and a flow rate of 10 μl/min. Rhosin (Millipore) and compounds JK-121~125 were dissolved in DMSO. Immobilized RHOA resulted in 2550 resonance units (RU). CLAMP^©^ program [46] was used to analyzed the kinetics of protein-small molecule binding.

## SUPPLEMENTARY MATERIALS DATA


