# Supplementary material for: Systematic approach identifies RHOA as a potential biomarker therapeutic target for Asian gastric cancer
Source: Oncotarget. 2016 Oct 28;7(49):81435–51. doi: 10.18632/oncotarget.12963 (PMC5348404; doi:10.18632/oncotarget.12963)
Supplement: Supplementary file 1 [file oncotarget-07-81435-s001.pdf]

Systematic approach identifies RHOA as a potential biomarker therapeutic target for Asian gastric cancer

SUPPLEMENTARY DATA

SUPPLEMENTARY METHODS

In Silico approach for identifying 730 RHOA small molecule compounds

Based on the "seed" ligand (the known RHOA inhibitor Rhosin), which molds the entire pocket of RHOA [18], we obtained similar candidate compounds for further docking and statistical analyses. We searched about 45 million compounds PUBCHEM, and found 730 similar backbone compounds with high Tanimoto scores of 0.6, measured by the Open Babel program [41].

Using these 730 chemical compounds, we performed docking simulations, based on the RHOA protein structure (Protein Data Bank entry ID 1X86 and chain B) [20]. The available docking pocket residues of the RHOA consisted of 75 residues. Docking simulation of each compound was performed as follows: i) Move the center of mass (COM) of a compound to an out-of-pocket residue; ii) Given that specific residue, perform an AutoDock Vina [42] calculation three times using different random seed; iii) Repeat steps 1 and 2 for all 75 pocket residues (i.e., a total number of docking executions= 3x75=225 for each compound). A 3D imaginary box bounded by equal dimensions of 15 Å was used to retain each ligand at a specifically assigned pocket residue. The center of the box was located on the Cα carbon of the pocket residue; iv) After docking simulations, the COMs of the conformations, within a radius of 4 Å, were clustered to generate multiple groups (i.e., clusters). When the two ligands approach each other within a radius of 4 Å, the two are assigned to the same group, as determined by CHARMM [43]. For each specific group, we assigned the lowest energy conformer as the representative conformer for that group. Presence of multiple groups for the same ligand suggests that the ligand is able to bind to different localities within the pocket, suggesting existence of nonspecific binding. For addressing this situation, we obtained the two groups (clusters) having the first (E_1) and second (E_2) lowest representative energies among the groups, and the two representative energy conformers were used for further analyses; and v) we then applied steps 1-4 to the remaining compounds (total number of docking executions: 730 x 225=164,250). After whole-docking simulations of all 730 compounds, we identified the group having the lowest representative energy conformer for each compound, resulting in 730 ligand conformers, as well as their corresponding energies.

Lipinski's rule application to the 730 compounds

Of the 730 compounds, the physicochemical properties relating to Lipinski's rule of five (RO5) [19], i.e., the number of hydrogen bond donors < 5, the number of hydrogen bond acceptors < 10, molecular mass < 500, octanol-water partition coefficient (logP) < 5, and the number of rotatable bonds < 10, were then applied as the second screen filter. Additionally, two more rules were applied: i) the number of heavy atoms ≤ 100; and ii) the number of compounds = 1. These two criteria reflect general properties of most currently used drugs, facilitating the selection of relatively small and moderately lipophilic molecules.

Since the candidate ligands could also interact with different local binding regions within the pocket of the RHOA protein, we assumed non-specific-RHOA binding as less preferable for druggability; consequently, aim to identify pocket-specific, locally binding ligands. For the purpose, we constructed a statistical test for evaluating druggability in terms of specific binding, which is described in the next section.

Specific binding test

Candidate ligand interactions with various local binding regions within the RHOA pocket can be shown by ligand-pocket clustering. In our hypothesis, higher ΔE values (difference between the lowest and the second lowest energies in the two binding regions) indicate that the compound is likely to specifically bind to the region having the lowest binding energy (Figure 8B). The compound in the right panel has specific binding to the pocket, compared to the left panel (Figure 8B). Considering that nonspecific protein-binding ligands were less preferable as druggable candidates, we developed a statistical test for identifying statistically significant, specific locally binding ligands.

For a given ligand candidate h $(h=1,..,m)$, we obtained the most stable ligand-pocket conformational state (state 1), and the second-most stable ligand-pocket conformational state (state 2), out of all the ligand-pocket pairs in the protein. We then defined the energy

corresponding to each conformational state i (i=1,2…) as E_{hi}, for each ligand h, using the postulate that the more ligand-specific RHOA binding, the higher the energy difference E_{h1}/E_{h2}. Using these parameters, we could establish a statistical measure for the significance of the energy differences.

We transformed E_{hi} (i=1,2) into exp(-E_{hi}/kT) ("C_{hi}"), defined as the occurrences of the ligand-pocket conformational state corresponding to E_{hi} (k: Boltzmann constant; T: temperature, with kT arbitrarily set to one). We then set C_{hi}~binomial(n_i, p_{hi}) (i=1,2), where n_i is the total number of occurrences for all E_{hi}s, and p_{hi} is the probability of the energy conformational state i for the ligand h. Using the relationship $X=\log_2 C_{h1}$, $Y=\log_2 C_{h2}$, $M=X-Y=\log_2(C_{h1}/C_{h2})$, and $A=(X+Y)/2$, and assuming that the C_{hi}s are independent and the n_is are large enough, the conditional distribution, M|A, follows a normal distribution that leads to the same statistical method used in DEGseq. Combining all of these considerations, we could obtain the significance of the energy differences for all the candidate compounds. The 41 most significant compounds by the tests were reported in Results section.

Small molecule synthesis

All reactions sensitive to air or moisture were conducted under nitrogen atmosphere. Reagents were purchased from Sigma-Aldrich and Tokyo Chemical Industry. All anhydrous solvents were distilled over CaH_2, P_2O_5, or Na/benzophenone prior to reaction, unless otherwise stated. Analytical thin-layer chromatography (TLC) was performed using commercial precoated TLC plates (Silicagel 60, F-254, Merck). Spots were detected by viewing under UV light (excitation, 254 nm), or colorizing with charring after dipping in any of the following solutions: phosphomolybdic acid (PMA) in ethanol or potassium permanganate aqueous solution. Flash column chromatography was performed on silica gel 60 (0.040~0.063 mm, 230-400 mesh, Merck). Infrared spectra were recorded using Agilent Cary670. ^{1}H NMR spectra ($CDCl_3$, CD_3OD, D_2O or DMSO-d_6) were recorded on Agilent 400-MR (400 MHz). The chemical shifts were reported in parts per million (δ) units, relative to the solvent peak. ^{1}H NMR data were reported as peak multiplicities: s for singlet; d for doublet; dd for doublet of doublets; ddd for doublet of doublet of doublets; t for triplet; pseudo t for pseudo triplet; brs for broad singlet; and m for multiplet. Coupling constants were reported in Hertz. ^{13}C NMR spectra ($CDCl_3$, CD_3OD, D_2O or DMSO-d_6) were similarly recorded using an Agilent 400-MR (100 MHz) device. The chemical shifts were reported as ppm (δ), relative to the solvent peak. Mass spectra were recorded on ESI^+ source in methylene chloride or methanol.

General synthesis procedures of hydrazides were as follows: A mixture of piperonal (7 mmol) and appropriate hydrazide compounds (7 mmol) in MeOH or EtOH were stirred at room temperature, or heated under reflux, for 2-40 h. The progress of reaction was monitored by TLC. After completion of reaction, contents were cooled to room temperature and poured into ice cold water (35 mL), while stirring. Solid was filtered, dried, and purified by recrystallization using MeOH or EtOH to give hydrazide products JK-121 ~ 125 in 64-95% yields.

Small molecular weight compound synthesis is as follows:

(E)-N'-(benzo[d][1,3]dioxol-5-ylmethylene) benzenesulfonohydrazide (JK-121): a yellow solid; IR (ATR) cm^{-1} 1624, 1594; ^{1}H NMR(400 MHz, DMSO-d_6) δ 11.37 (s, 1H), 7.87 (d, J = 6.8 Hz, 2H), 7.81 (s, 1H), 7.62 (m, 3H), 7.09 (d, J =1.4 Hz, 1H), 7.02 (dd, J = 1.4, 8 Hz, 1H), 6.92 (d, J = 8 Hz, 1H), 6.05 (s, 2H); ^{13}C NMR (100 MHz, DMSO-d_6) δ 149.06, 147.87, 147.04, 138.96, 133.01, 129.01, 127.99, 127.20, 123.02, 108.39, 104.86, 101.53; HRMS calcd for $C_{14}H_{12}N_2O_4S$ $[M+H]^+$ 305.0596; found 305.0596

(E)-N'-(benzo[d][1,3]dioxol-5-ylmethylene) benzohydrazide (JK-122): a white solid; IR (ATR) cm^{-1} 1654, 1627, 1593; ^{1}H NMR(400 MHz, DMSO-d_6) δ 11.75 (s, 1H), 8.37 (s, 1H), 7.89 (d, J = 7.3 Hz, 2H), 7.54 (m, 3H), 7.31 (s, 1H), 7.17 (d, J= 7.8, 1H), 7.00 (d, J = 7.9 Hz, 1H), 6.06 (s, 2H); ^{13}C NMR (100 MHz, DMSO-d_6) δ 162.97, 149.10, 147.99, 147.58, 133.51, 131.66, 128.74, 128.45, 127.56, 123.34, 108.49, 105.10, 101.58; HRMS calcd for $C_{15}H_{12}N_2O_3$ $[M+H]^+$ 269.0926; found 269.0927

(E)-N'-(benzo[d][1,3]dioxol-5-ylmethylene) isonicotinohydrazide (JK-123): a white solid; IR (ATR) cm^{-1} 1785, 1675, 1626; ^{1}H NMR(400 MHz, DMSO-d_6) δ 11.97 (s, 1H), 8.78 (dd, J = 1.4, 4.5 Hz, 2H), 8.37 (s, 1H), 7.82 (dd, J = 1.4, 4.5 Hz, 2H), 7.31 (s, 1H), 7.20 (dd, J = 1.4, 8 Hz, 1H), 7.01 (d, J = 8 Hz, 1H), 6.11(s, 2H); ^{13}C NMR (100 MHz, DMSO-d_6) δ 161.43, 150.30, 149.36, 148.78, 148.03, 140.57, 128.41, 123.67, 121.50, 108.53, 105.20, 101.63; HRMS calcd for $C_{14}H_{11}N_3O_3$ $[M+H]^+$ 270.0879; found 270.0896

(*E*)-*N'*-(benzo[*d*][1,3]dioxol-5-ylmethylene)-4-hydroxybenzohydrazide (JK-124): a white solid; IR (ATR) cm^{-1} 1783, 1648, 1595; ^{1}H NMR(400 MHz, DMSO-d_6) δ 11.54 (s, 1H), 10.09 (s, 1H), 8.33 (s, 1H), 7.78 (d, J = 8.6 Hz, 2H), 7.28 (s, 1H), 7.14 (d, J = 8.0 Hz, 1H), 6.98 (d, J = 8.0 Hz, 1H), 6.84 (d, J = 8.6 Hz, 2H),

6.09 (s, 2H); ^{13}C NMR (100 MHz, DMSO-d_6) δ 162.60, 160.64, 148.90, 147.96, 146.61, 129.58, 128.95, 123.90, 123.07, 114.98, 108.46, 105.01, 101.51; HRMS calcd for $C_{15}H_{12}N_2O_4$ $[M+H]^+$ 285.0875; found 285.0877.

(E)-N'-(benzo[d][1,3]dioxol-5-ylmethylene) acetohydrazide (JK-125): a white solid; IR (ATR) cm^{-1} 1757, 1677, 1597; ^{1}H NMR(400 MHz, DMSO-d_6) δ 11.13 (s, 1H), 7.88 (s, 1H), 7.24 (d, J = 1.4, 1H), 7.07 (dd, J = 1.4, 8 Hz, 1H), 6.94 (d, J = 8 Hz, 1H), 6.07(s, 2H), 2.17 (s, 3H); ^{13}C NMR (100 MHz, DMSO-d_6) δ 171.79, 148.67, 147.92, 142.19, 128.75, 122.66, 108.41, 104.85, 101.45, 20.26; HRMS calcd for $C_{10}H_{10}N_2O_3$ $[M+H]^+$ 207.0770; found 207.0766

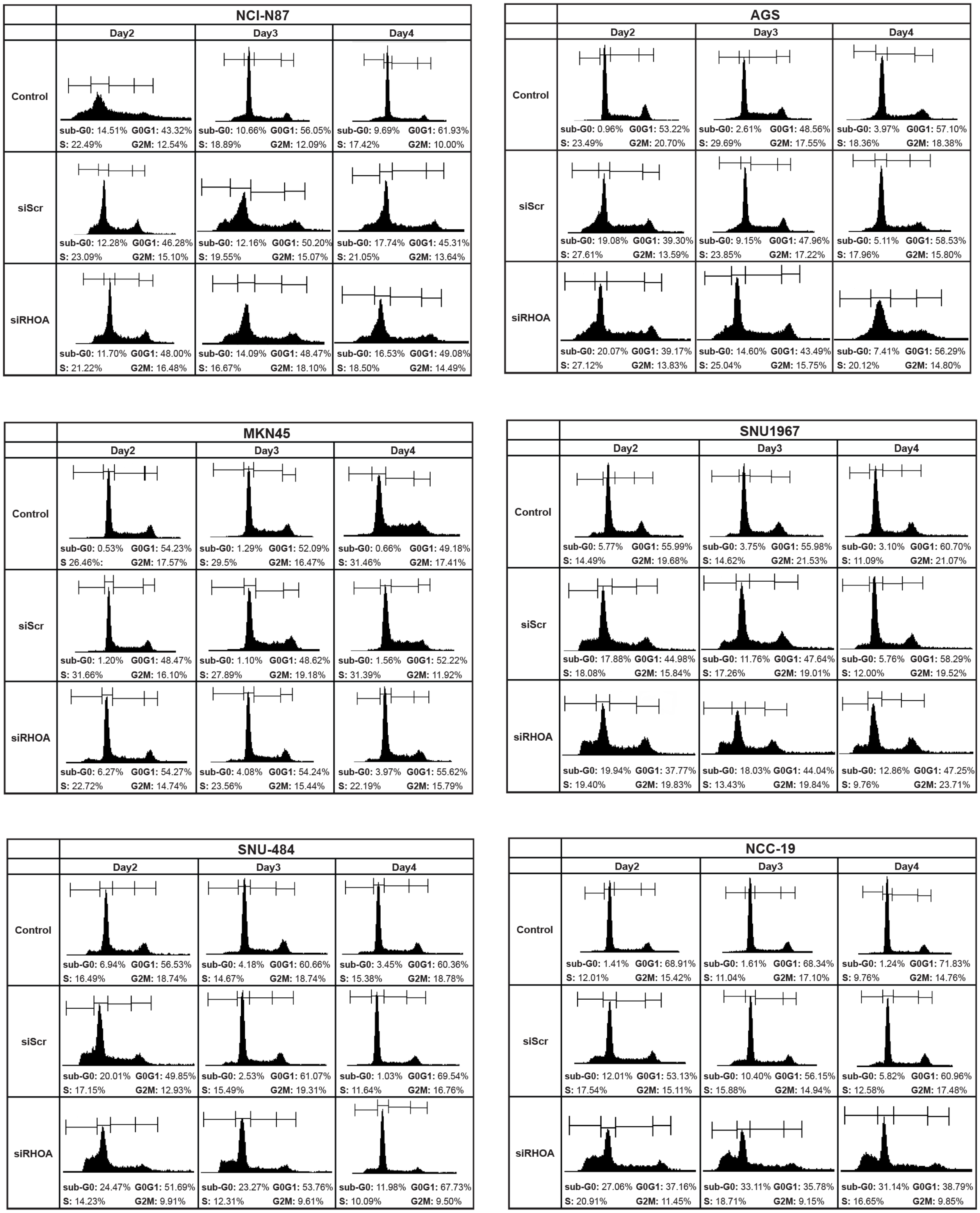

Supplementary Figure S1: After siRNA knockdown of *RHOA*, cell cycle was analyzed by flow cytometry. For NCI-N87 GC cells, the respective cell phase populations were similar between *RHOA* knockdown cells versus control cells. AGS cells showed sensitivity to the transfection reagent (data not shown), and little difference between cell cycle phase were observed between siScr and siRHOA, similar to SNU-1967. For MKN45, NCC-19 and SNU-484, increased a sub-G0 population was observed when *RHOA* was knocked down.

Supplementary Table S1: The sample information of omics datasets in the study. We described the sample information including the number of samples in each stage

Dataset	Asian						Caucasian					
	Normal	Stage I	Stage II	Stage III	Stage IV	Missing	Normal	Stage I	Stage II	Stage III	Stage IV	Missing
GSE36968 (Figure 1B)	6	5	5	8	6	0	N/A	N/A	N/A	N/A	N/A	N/A
TCGA (Figure 2A, Figure 6C)	6	7	36	31	3	0	17	18	55	77	6	16
